# A Network-Based Approach to Visualize Prevalence and Progression of Metabolic Syndrome Components

**DOI:** 10.1371/journal.pone.0039461

**Published:** 2012-06-19

**Authors:** Robin Haring, Martin Rosvall, Uwe Völker, Henry Völzke, Heyo Kroemer, Matthias Nauck, Henri Wallaschofski

**Affiliations:** 1 Institute of Clinical Chemistry and Laboratory Medicine, University Medicine Greifswald, Greifswald, Germany; 2 Department of Physics, Umeå University, Umeå, Sweden; 3 Department of Functional Genomics, University Medicine Greifswald, Greifswald, Germany; 4 Institute for Community Medicine, University Medicine Greifswald, Greifswald, Germany; 5 Institute of Pharmacology, University Medicine Greifswald, Greifswald, Germany; Inrca, Italy

## Abstract

**Background:**

The additional clinical value of clustering cardiovascular risk factors to define the metabolic syndrome (MetS) is still under debate. However, it is unclear which cardiovascular risk factors tend to cluster predominately and how individual risk factor states change over time.

**Methods & Results:**

We used data from 3,187 individuals aged 20–79 years from the population-based Study of Health in Pomerania for a network-based approach to visualize clustered MetS risk factor states and their change over a five-year follow-up period. MetS was defined by harmonized Adult Treatment Panel III criteria, and each individual's risk factor burden was classified according to the five MetS components at baseline and follow-up. We used the map generator to depict 32 (2^5^) different states and highlight the most important transitions between the 1,024 (32^2^) possible states in the weighted directed network. At baseline, we found the largest fraction (19.3%) of all individuals free of any MetS risk factors and identified hypertension (15.4%) and central obesity (6.3%), as well as their combination (19.0%), as the most common MetS risk factors. Analyzing risk factor flow over the five-year follow-up, we found that most individuals remained in their risk factor state and that low high-density lipoprotein cholesterol (HDL) (6.3%) was the most prominent additional risk factor beyond hypertension and central obesity. Also among individuals without any MetS risk factor at baseline, low HDL (3.5%), hypertension (2.1%), and central obesity (1.6%) were the first risk factors to manifest during follow-up.

**Conclusions:**

We identified hypertension and central obesity as the predominant MetS risk factor cluster and low HDL concentrations as the most prominent new onset risk factor.

## Introduction

The metabolic syndrome (MetS) has gained recent attention as a multifactorial cluster of cardiovascular risk factors linked to criteria of adiposity, hypertension, dyslipidemia, and hyperglycaemia. Although associations between MetS and incident cardiovascular disease (CVD) have been repeatedly observed [1], there is an ongoing controversy about its clinical value and impact [2]–[6]. Compared to established CVD risk scores, MetS is a relatively weak predictor of incident CVD and its single components showed a comparable or even better predictive utility, respectively [7]–[9]. We previously showed that there was no added predictive value of MetS beyond its individual components with respect to mortality risk [10] and concluded in line with others to redirected attention to its individual components, particularly central obesity and hyperglycaemia [10], [11]. Also with regard to cardiovascular risk, MetS was admittedly associated with accelerated central arterial ageing, but specific clusters of MetS components showed dramatically increased arterial changes [12]. But although these outcome associations may give a clue which risk factors drive the underlying MetS pathophysiology, investigations which MetS components tend to cluster predominately and how individual risk factor states change over time are scarce [13], [14]. Therefore, we used a novel network-based approach to visualize clustered MetS components and their change during a five-year follow-up period among 3,187 participants from the population-based Study of Health in Pomerania (SHIP).

## Methods

### Ethics statement

The study protocol was consistent with the principles of the Declaration of Helsinki and approved by the local Ethics Committee of the University of Greifswald.

### Study population

The SHIP is a population-based cohort study conducted in Northeast Germany. Details about the study's sampling method, design, and exams were published previously [15]. A representative sample comprising of 7,008 individuals was selected using population registries where all German inhabitants are registered. Only individuals with German citizenship and main residency in the study area were included. The net sample (without migrated or deceased persons) comprised 6,267 eligible individuals. At baseline, SHIP finally comprised 4,308 (2,116 men) participants (response 68.8%) with examinations conducted between 1997 and 2001. Between 2002 and 2006, all participants were re-invited to a five-year follow-up examination, and 3,300 individuals (1,589 men) participated (response 83.5%). All participants gave written informed consent. Of the 3,300 individuals attending SHIP baseline and follow-up examinations we excluded individuals with missing data on MetS components, analyzing a final study population of 3,187 individuals (1,546 men).

### Measures

Baseline and follow-up examinations included a computer-assisted personal interview, as well as somatometric, medical, and laboratory measurements. Waist circumference (WC) was measured to the nearest 0.1 cm using an inelastic tape midway between the lower rib margin and the iliac crest in the horizontal plane, with the subject standing comfortably with weight distributed evenly on both feet. After a resting period of at least five minutes, systolic and diastolic blood pressure was measured three times in the right arm of seated participants using a digital blood pressure monitor (HEM-705CP, Omron Corporation, Tokyo, Japan) with each reading being followed by a three-minute pause. The second and third readings were averaged to give the mean diastolic and systolic blood pressure.

Non-fasting blood samples were taken from the cubital vein in the supine position between 7:00 a.m. and 7:00 p.m. and prepared for immediate analysis or for storage at −80°C for further analysis. Serum high-density lipoprotein (HDL) cholesterol concentrations were measured photometrically at baseline (Hitachi 704, Roche, Mannheim, Germany), whereas follow-up HDL concentrations were quantified by lipid electrophoresis (HELENA SAS-3 system, Helena 7 BioSciences Europe, Tyne & Wear, UK). To ensure comparability in the longitudinal HDL analyses, we used baseline HDL concentrations as the reference and calculated corrected follow-up HDL concentrations based on a previously published conversion formula [16]. Doing so, we found that the average HDL concentrations produced by the two methods were virtually identical, suggesting that the differences in HDL will be small within the range of practical relevance [17]. Serum triglyceride and glucose concentrations were determined enzymatically using reagents from Roche Diagnostics (Hitachi 717, Roche Diagnostics, Mannheim, Germany). All assays were performed according to the manufacturers' recommendations by skilled technical personnel, and internal quality controls were analyzed daily. In addition, the laboratory participates in official quarterly German external proficiency testing programs. Type 2 diabetes mellitus was defined based on self-reported physicians diagnosis or use of antidiabetic medication (anatomic-therapeutical-chemical [ATC] code A10) in the last seven days, or glycated haemoglobin (HbA1c) concentrations >48.0 mmol/mol. CVD was defined one or more components of a previously published summative score comprising information about peripheral artery disease, heart failure, angina pectoris, and a recall of physician's diagnoses of stroke and myocardial infarction [18].

Diagnostic criteria for the assessment of MetS components were defined according the Joint Scientific Statement to harmonize MetS [19] and modified for the use of non-fasting blood samples, as previously established in SHIP [20], [21] and other large cohorts [22], [23]:

1. elevated WC: men >94 cm, women >80 cm;
2. elevated non-fasting glucose: ≥8.0 mmol/l or antidiabetic treatment (ATC code A10A);
3. decreased HDL cholesterol: men <1.0 mmol/l, women <1.3 mmol/l, or lipid-lowering treatment (ATC C10AB, A10AD);
4. elevated non-fasting triglycerides: ≥2.3 mmol/l or lipid-lowering treatment (ATC C10AB, A10AD);
5. elevated blood pressure: ≥130/85 mmHg or antihypertensive drug treatment (ATC codes C02, C03, C04, C07, C08, C09). Participants fulfilling at least three out of these five components were assigned to MetS.

### Statistical Analysis

**Figure 1:**
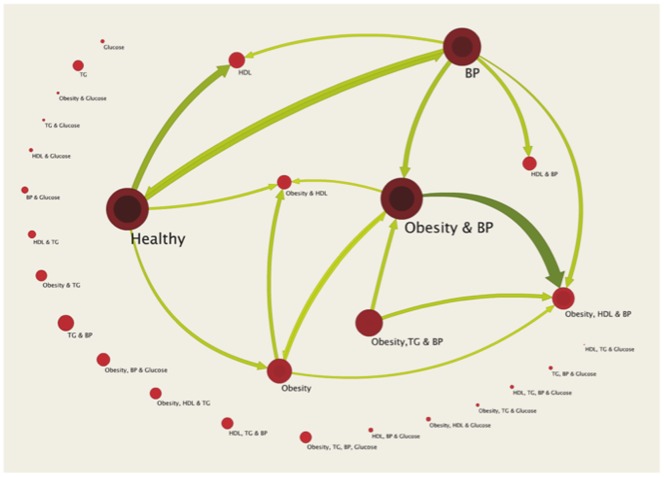
Metabolic syndrome network visualizing cardiovascular risk factor burden, clustering, and flow of its components between baseline and five-year follow-up. “**Healthy**”, no prevalent metabolic syndrome component; “**Obesity**”, waist circumference: men >94 cm, women > 80 cm; “**Glucose**”, elevated non-fasting glucose: ≥8.0 mmol/l or antidiabetic treatment (anatomic-therapeutical-chemical [ATC] codes A10A, A10B); “**HDL**”, decreased high-density lipoprotein cholesterol: men <1.0 mmol/l, women <1.3 mmol/l, or lipid-lowering treatment (ATC C10AB, A10AD); “**TG**”, elevated non-fasting triglycerides: ≥2.3 mmol/l or lipid-lowering treatment (ATC C10AB, A10AD); “**BP**”, elevated blood pressure: ≥130/85 mmHg or antihypertensive drug treatment. The size of a node is proportional to the number of individuals in the risk factor state that the node represents. The inner circle of a node denotes individuals remaining in that risk factor state between baseline and follow-up, whereas the outer circle of a node denotes individuals moving between risk factor states. The networks' flow between baseline and follow-up is shown for the 39 most important links (13% of all 301 links), representing 84.5% of total flow. The weight and colour shade of a link represent the number of individuals moving between the two states that the link connects.

Categorical data are reported as percentages, and continuous data are reported as median together with the interquartile range. The individual risk factor burden was classified to states according to the five MetS components at baseline and follow-up. With five MetS components, there are 2^5^ = 32 different states and 32^2^ = 1,024 possible transitions between states from baseline to follow-up. We represented this data as a weighted directed network with states as nodes and transitions between states as weighted directed links between the nodes. We used the map generator at http://www.mapequation.org/mapgenerator/ to generate a map of risk factor flow between the potential risk factor states, including the state “healthy” with absence of any MetS risk factors. **Figure 1** shows the risk factor map with link width and intensity proportional to the weight of the links and node size proportional to the number of individuals assigned to it at baseline. The inner circle of a node represents the number of individuals that remain in that state between baseline and follow-up (self-links), and the outer ring represents the number of individuals that transits to a different risk factor state. To evaluate potential non-response bias due to drop out between baseline and the five-year follow-up examination, baseline characteristics of follow-up responder vs. non-responder were compared using χ^2^ tests for categorical data or two-sample t-tests for continuous data. As expected, we observed that follow-up non-responder were on average older, male, and exposed adverse cardiometabolic risk factor profiles compared to follow-up responder, but found no differences in MetS prevalence, the main outcome of the present study. Data preparation and descriptive statistics were performed with Stata 11.0 (Stata Corp., College Station, TX, USA).

## Results

Characteristics of the study population at baseline and follow-up are presented in **Table 1**. MetS prevalence at baseline was 27.6% and 42.4% after a median follow-up time of 5.0 years, respectively. The risk factor network in **Figure 1** shows the baseline risk factor profiles of 3,187 individuals and how their profiles change during the follow-up period. For visual simplicity, the depicted network only shows the 28 most prevalent profiles, representing 91% of all possible risk factor states at baseline. For the same reason, we have only included the 39 strongest connections (13% of links), but nevertheless captured 85% of all risk factor flow. At baseline, we found the largest fraction (19.3% of all individuals) in the healthy state without any MetS risk factors, of which 58% remained risk factor free and 42% developed risk factors at follow-up (11.1% of all individuals were without any MetS risk factors at baseline and follow-up) (**Table 2**). We identified hypertension (15.4% “BP”) and central obesity (6.3% “Obesity”), as well as their combination (19.0%) as the most common MetS risk factors. In **Table 2** we present detailed information about the most common risk factor states that each comprised more than 5% of the overall sample. The largest risk factor flow in terms of number of individuals was the transition from hypertension and central obesity to additional low HDL concentrations at follow-up (6.3%). The largest risk factor flows from the healthy state were to low HDL concentrations (3.5%), hypertension (2.1%), and central obesity (1.6%) (**Table 3**). But in general, most individuals remained in their risk factor state, and self-links dominated the risk factor flow network during the five-year follow-up (**Table 3**).

**Table 1:** Baseline and follow-up characteristics of the study population (N = 3,187).

Characteristic	Baseline	Follow-up	p-value
Age, years	50.0 (37.1, 61.6)	55.0 (42.2, 66.4)	<0.001
Sex (women), %	51.5	N.A.	
Waist circumference, cm	89.0 (78.8, 98.5)	92.4 (82.5, 102.0)	<0.001
Diastolic blood pressure, mmHg	83.0 (76.0, 90.5)	81.0 (74.0, 88.0)	<0.001
Systolic blood pressure, mmHg	134.0 (121.0, 148.0)	131.0 (119.0, 144.0)	<0.001
High-density lipoprotein cholesterol (HDL-C), mmol/l	1.4 (1.2, 1.7)	1.1 (0.9, 1.4)	<0.001
Total cholesterol, mmol/l	5.7 (4.9, 6.5)	5.5 (4.7, 6.3)	<0.001
Low-density lipoprotein cholesterol, mmol/l	3.5 (2.8, 4.2)	3.5 (2.8, 4.2)	0.014
Triglyceride, mmol/l	1.5 (1.0, 2.3)	1.5 (1.0, 2.2)	0.895
Serum glucose, mmol/l	5.3 (4.9, 5.8)	5.2 (4.8, 5.7)	<0.001
Haemoglobin A1c, mmol/mol	34 (30, 39)	34 (30, 39)	0.447
Antidiabetic medication, %	5.2	8.4	<0.001
Type 2 diabetes mellitus, %	10.5	12.8	<0.001
Antihypertensive medication, %	22.6	36.0	<0.001
Lipid-lowering medication, %	7.5	14.5	<0.001
Cardiovascular disease, %	16.3	14.6	<0.001
Metabolic syndrome, %	27.6	42.4	<0.001
Elevated waist circumference, %	54.4	65.2	<0.001
Elevated glucose, %	6.7	9.9	<0.001
Decreased HDL-C, %	23.9	57.3	<0.001
Elevated triglycerides, %	25.1	22.8	<0.001
Elevated blood pressure, %	65.1	65.5	<0.001

Data are presented as percentages or median (25^th^ and 75^th^ percentile).

* p-values based the χ^2^ test (categorical data) or T-test (continuous data).

**Table 2:** Most dominant risk factor cluster at baseline (>5% of individuals).

Rank	Risk factor cluster	Baseline (%)	Follow-up self-links (%)	Follow-up out-flow (%)
1	Healthy	19.3	8.2	11.1
2	Obesity & BP	19.0	6.9	12.1
3	BP	15.4	4.7	10.8
4	Obesity, TG & BP	7.7	0.5	7.2
5	Obesity	6.3	1.5	4.8
6	Obesity, HDL, TG & BP	6.2	2.9	3.3
7	Obesity, HDL & BP	5.1	2.4	2.7

Follow-up self-links refers to the proportion of individuals who did not change their baseline risk factor profile at follow-up. Follow-up out-flow refers to the proportion of individuals who did change their baseline risk factor profile at follow-up.

**Table 3:** Risk factor flow for the 39 most important links.

Rank	Risk factor cluster from baseline to follow-up	Change in number of MetS components	Total flow (%)
1	Healthy Healthy	=	8.2
2	Obesity & BP Obesity & BP	=	6.9
3	Obesity & BP Obesity, HDL & BP	+	6.3
4	BP BP	=	4.6
5	Healthy HDL	=	3.5
6	Obesity, HDL, TG, BP Obesity, HDL, TG & BP	=	2.9
7	Obesity, TG & BP Obesity, HDL, TG & BP	+	2.6
8	Obesity, HDL & BP Obesity, HDL & BP	=	2.5
9	BP Obesity & BP	+	2.2
10	Healthy BP	=	2.1
11	Obesity & BP Obesity, HDL, TG & BP	+	1.9
12	BP HDL & BP	+	1.8
13	BP Obesity, HDL & BP	+	1.8
14	BP Healthy	=	1.7
15	Obesity Obesity & HDL	+	1.7
16	Healthy Obesity	=	1.6
17	Obesity, TG & BP Obesity, HDL & BP	=	1.6
18	Obesity Obesity	=	1.5
19	Obesity, TG & BP Obesity & BP	−	1.5
20	Obesity, HDL, TG & BP Obesity, HDL & BP	−	1.2
21	HDL HDL	=	1.1
22	Healthy Obesity & HDL	+	0.9
23	Obesity Obesity & BP	+	0.9
24	BP HDL	=	0.9
25	Obesity, HDL & BP Obesity, HDL, TG, & BP	+	0.9
26	Obesity Obesity, HDL & BP	+	0.9
27	Obesity, HDL, TG, BP & Glucose Obesity, HDL, TG, BP & Glucose	=	0.8
28	Obesity & BP Obesity	−	0.8
29	Obesity & HDL Obesity & HDL	=	0.7
30	HDL & BP HDL & BP	=	0.7
31	Obesity, HDL, TG & BP Obesity, HDL, TG, BP & Glucose	+	0.6
32	Obesity, BP & Glucose Obesity, HDL, BP & Glucose	+	0.6
33	BP HDL, TG & BP	+	0.6
34	Obesity & BP Obesity & HDL	=	0.6
35	Obesity, TG, BP & Glucose Obesity, HDL, TG, BP & Glucose	+	0.6
36	Healthy Obesity & BP	+	0.5
37	Obesity & HDL Obesity, HDL & BP	+	0.5
38	Healthy Obesity, HDL & BP	+	0.5
39	Obesity, TG & BP Obesity, TG & BP	=	0.5

These 39 most important links represent 13% of all 301 links covering 84.5% of the total flow.

“Obesity”, waist circumference: men >94 cm, women >80 cm;

“Glucose”, elevated glucose: ≥8.0 mmol/l or antidiabetic treatment;

“HDL”, decreased high-density lipoprotein cholesterol: men <1.0 mmol/l, women <1.3 mmol/l, or lipid-lowering treatment;

“TG”, elevated non-fasting triglycerides: ≥2.3 mmol/l or lipid-lowering treatment;

“BP”, elevated blood pressure: ≥130/85 mmHg or antihypertensive drug treatment.

## Discussion

The present network-based analyses of longitudinal data from 3,187 individuals identified hypertension and central obesity as the most common MetS components. These MetS components also constituted the predominant risk factor cluster during follow-up. Analyzing risk factor change, we revealed low HDL concentrations as the most common additional and new onset MetS component. This is the first network-based approach to predictively model cardiovascular risk factor burden and expression. To better understand which MetS components tend to cluster predominately and how individual risk factor states change over time, we not only explored the topology of the MetS risk factor network, but also its dynamic changes over a five-year follow-up period.

Although the MetS network contains a plethora of potentially interconnected cardiovascular risk factor states, only some are truly relevant to the pathophenotype MetS and its suggested outcome associations with CVD and mortality [12], [24]–[27]. The commonly as most relevant MetS components considered cardiovascular risk factors are central obesity and insulin resistance. But although insulin resistance is the hallmark of MetS, central obesity is the most relevant predisposing factor for insulin resistance [28]. Thus, the finding of a predominant role of central obesity, but not glycaemia, in our MetS network is likely explained by our study sample consisting of individuals selected from the general population, and was also observed in previous population studies [13], [14] because in contrast to patient populations, there is a large proportion of individuals without any MetS components and a relatively low cardiovascular risk factor burden.

Obesity is becoming a global epidemic, with a continued worldwide trend of increasing prevalences over the past four decades [29]. Its public health impact stems from the notion that obesity raises CVD risk through other risk factors including dyslipidemia, hypertension, and hyperglycemia, and is therefore the underlying risk factor in the pathogenesis of CVD [30]. For example, the INTERHEART study examined more than 29,000 individuals in 52 countries to show that more than 90% of the risk for acute myocardial infarction is predicted by nine traditional risk factors including hypertension and central obesity [31]. In a substudy of Latin American INTERHEART countries, central obesity was the most important population-attributable risk factor for acute myocardial infarction [32]. Using a different statistical approach, a six-year follow-up of 506 men and 461 women from the Baltimore Longitudinal Study on Aging identified higher baseline abdominal obesity or triglycerides, and lower HDL cholesterol as predictors of incident MetS [13]. Thus, despite varying definitions of the MetS [19] and study samples, central obesity was identified as a key component showing links between the other MetS components [33].

The central role of hypertension in our MetS network analysis is in line with a previous longitudinal investigation from the Framingham Heart Study that found hypertension as the risk factor most often associated with MetS diagnosis [14]. Similarly, prior data from the National Health and Nutrition Examination Survey (NHANES) showed that hypertension was the most common MetS component in men and third most common in women [34]. A recent Principal Components Analysis of MetS risk factors, also identified elevated blood pressure as the most common MetS component [35]. But interestingly, the MetS components with the highest prevalence prior to MetS development, such as elevated blood pressure, are not necessarily the strongest risk factors of incident MetS [13]. However, despite the comparably high prevalence of hypertension within our study region [36], these findings from different study populations and geographic regions limit the potential impact of regional differences on our revealed estimates.

Taken together, the predominant cardiovascular risk factor cluster identified in the present MetS network analysis consists of hypertension and central obesity. Our results suggest assessing single cardiovascular risk factors as a simpler alternative to MetS for CVD risk identification [10], especially in the general population, before other MetS components appear clinically. Similarly, the Framingham Heart Study identified a risk factor combination of central obesity, hypertension, and hyperglycemia, which more than doubled the risk of incident CVD and mortality [14]. Analyzing trajectories of entering the MetS, central obesity was also identified to confer the highest risk of incident MetS [14].

We showed that the contribution of the various cardiovascular risk factors to the MetS individually and collectively is unbalanced and therefore requires proper identification to provide adequate treatment. To avoid obesity-initiated MetS and the downstream clustering of additional cardiovascular risk factors like hypertension or dyslipidemia, lifestyle interventions that include increased physical activity and dietary modifications offer an evidence-based strategy for managing obesity [29], as well as prehypertension and prediabetes [37]. Cardio-respiratory fitness has also been identified as a key explanatory variable for the risks associated with obesity and MetS. After inclusion of a variable reflecting cardio-respiratory fitness into a statistical model predicting all-cause and CVD mortality from MetS and obesity, the risk estimates were no longer significant [38]. However, the fundamental challenge remains how to intervene at the public health level to address the obesity epidemic in the general population. The interventions proven to be cost-effective and to prevent the transition to MetS should be prioritized for implementation.

### Strengths and limitations

Strengths of the present investigation include the high-quality longitudinal data from a large-scale population-based epidemiological cohort and its network-based visualization. Limitations may arise from the use of non-fasting blood samples for the diagnosis of ATP III defined MetS. But due to logistical concerns in a large-scale population-based study like SHIP, it was practically impossible to obtain such. However, the applied MetS definition, based on non-fasting blood samples, was published in several previous investigations related to MetS from our cohort [10], [20], [21], [39] and was suggested to be even the better surrogate for the diagnosis of MetS, in particular for population-based epidemiological studies like the present [40]. Furthermore, intra-individual variation in the repeatedly assessed outcome measures (including non-fasting triglycerides or glucose) may have caused classification bias whose extent we were not able to evaluate using network-based descriptive statistical analyses. However, given the large sample size and the repeated one-point measurements of MetS components, potential intra-individual variability is supposed to cause misclassification into both directions.

### Conclusions

Harvesting a network-based approach to visualize cardiovascular risk factor burden, expression, and change, we identified hypertension and central obesity as the predominant MetS risk factor cluster and low HDL concentrations as the most prominent new onset risk factor. Interestingly, the identified subnetwork or risk factor cluster has been shown to overlap within similar pathophysiological processes finally leading to MetS, overt clinical CVD, and mortality. However, prolonged efforts are needed to identify high-risk individuals and to provide them with effective evidence-based therapies. By revealing further insights into the onset and progression of MetS risk factors, we have contributed to the successful application of systems principles in epidemiology [41], a new field tentatively named network medicine [42], [43].
